# Referrals to liaison services for older adults with deliberate self harm during the SARS-CoV-2 national lockdown - a collaborative service evaluation using liaison referral data

**DOI:** 10.1192/bjo.2021.140

**Published:** 2021-06-18

**Authors:** Josie Jenkinson, Kehinde Junaid, Sara Ormerod, Sunita Sahu, Hugh Grant-Peterkin, Mazen Daher, James Lee-Davey, Atilla Yetkil, Julian Beezhold, Adrian Leddy, Elizabeth Sampson, Tasnia Chowdhury, Bushra Babar, Parrthiepan Visvaratnam, Divya Vamathevan, Rogin Deylami, Tristan Sawle, Mollie Delaney, Ahoane Qureshi, Rabeya Rahman, Neelam Sharma, Kareem Pabani, Jack Hubbett, Yuki Takao, Ellie Hanton

**Affiliations:** 1 Surrey and Borders Partnership NHS Foundation Trust; 2 Nottinghamshire Healthcare NHS FT; 3 Birmingham and Solihull MH NHS FT; 4 Oxleas NHS FT; 5 East London NHS FT; 6 Norfolk and Suffolk NHS FT; 7 Barnet Enfield and Haringey MH Trust

## Abstract

**Aims:**

Social isolation and living alone have been associated with increased suicidality in older adults. During the SARS-CoV-2 pandemic, older adults were advised to keep isolated and maintain social distancing. Lockdown periods in England may have led to increased isolation and loneliness in older people, possibly resulting in an increased rates of DSH and suicide. This study aimed to explore whether numbers of older adults referred to liaison services with deliberate self harm changed during the SARS-CoV-2 pandemic.

**Method:**

Reason for referral and total number of referrals to liaison services for older adults data were collected across 6 mental health trusts who had access to robust data sets. Data were collected prospectively for three months from the start of the UK national lockdown and for the corresponding 3 month period in 2019, via trust reporting systems. This study was registered as service evaluation within each of the participating mental health trusts.

**Result:**

Overall numbers of referrals to older adult liaison services went down, but the proportion of referrals for older adults with DSH increased. Across the six mental health trusts there there were a total of 2167 referrals over the first three month lockdown period in 2020, and 170 (7.84%) of these referrals were for deliberate self harm. During a corresponding time period in 2019, there were a total of 3416 referrals and 155 (4.54%) of these referrals were for deliberate self harm

**Conclusion:**

Although numbers of referrals for older adults with delberate self harm appeared to stay the same, the severity of these presentations is not clear. Outcomes of referrals and severity of self harm could be explored by examining individual case records. As there have been subsequent lockdowns the data collection period should also be extended to include these. Triangulation with national and local datasets on completed suicide is planned.

